# 肺癌合并慢阻肺患者术后运动康复获益探究

**DOI:** 10.3779/j.issn.1009-3419.2021.102.51

**Published:** 2022-01-20

**Authors:** 中华 余, 国省 谢, 昌龙 秦, 小明 邱

**Affiliations:** 610000 成都，四川大学华西医院 West China Hospital, Sichuan University, Chengdu 610000, China

**Keywords:** 肺肿瘤, 术后康复, 运动耐力, 肺功能, 微信计步, Lung neoplasms, Postoperative rehabilitation, Exercise endurance, Lung function, We-chat step counting

## Abstract

**背景与目的:**

慢性阻塞性肺病（chronic obstructive pulmonary diseases, COPD）影响全世界范围内45%-63%的肺癌患者，合并COPD的肺癌患者心肺功能降低，围术期风险增加，且术后运动耐力和肺功能较常规肺癌患者下降幅度更大。已有研究表明术后运动训练可以改善未经选择的肺癌患者运动耐力，但合并COPD的肺癌患者是否也能从术后运动训练中获益尚不清楚，本研究拟探究术后运动训练对肺癌合并COPD患者运动耐力、日常活动能力和肺功能的影响。

**方法:**

前瞻性分析2020年8月5日-2021年8月25日于四川大学华西医院肺癌中心行肺切除术治疗的非小细胞肺癌（non-small cell lung cancer, NSCLC）合并COPD患者74例，随机分为运动组和对照组；两组患者术后第1周接受常规术后康复，从第2周开始对照组给予常规护理，运动组在此基础上进行术后运动康复训练，为期2周。术前3天进行基线评估，术后3周进行终点评估。

**结果:**

两组患者的运动耐力，日常活动能力和肺功能测试结果均从基线到终点有所下降，但在手术和干预方案后，运动组心肺运动试验的最大耗氧量和6分钟步行试验的步行距离均显著优于对照组[(13.09±1.46) mL/kg/min *vs* (11.89±1.38) mL/kg/min, *P*=0.033; (297±46) m *vs* (243±43) m, *P*=0.041]。运动组平均微信计步数量也显著优于对照组（4, 381±397 *vs* 3, 478±342, *P*=0.035）。运动组用力肺活量（forced vital capacity, FVC）和一秒用力呼气容积（forced expiratory volume in one second, FEV_1_）均优于对照组，但差异未达到统计学显著水平[(1.76±0.19) L *vs* (1.60±0.28) L, *P*=0.084; (1.01±0.17) L *vs* (0.96±0.21) L, *P*=0.467]。

**结论:**

术后运动康复训练可以改善肺癌合并COPD患者运动耐力和日常活动能力，促进术后康复。

## Abstract (en)

**Background and objective:**

Chronic obstructive pulmonary diseases (COPD) affects 45%-63% of lung cancer patients worldwide. Lung cancer patients complicated with COPD have decreased cardiopulmonary function and increased perioperative risk, and their postoperative exercise endurance and lung function are significantly lower than those with conventional lung cancer. Previous studies have shown that postoperative exercise training can improve the exercise endurance of unselected lung cancer patients, but it is unclear whether lung cancer patients with COPD can also benefit from postoperative exercise training. This study intends to explore the effects of postoperative exercise training on exercise endurance, daily activity and lung function of lung cancer patients with COPD.

**Methods:**

Seventy-four patients with non-small cell lung cancer (NSCLC) complicated with COPD who underwent pneumonectomy in the lung cancer center of West China Hospital of Sichuan University from August 5, 2020 to August 25, 2021 were prospectively analyzed. They were randomly divided into exercise group and control group; The patients in the two groups received routine postoperative rehabilitation in the first week after operation, and the control group was given routine nursing from the second week. On this basis, the exercise group received postoperative exercise rehabilitation training for two weeks. Baseline evaluation was performed 3 days before operation and endpoint evaluation was performed 3 weeks after operation.

**Results:**

The exercise endurance, daily activity and pulmonary function test results of the two groups decreased from baseline to the end point. However, after the operation and intervention program, the maximum oxygen consumption of Cardiopulmonary Exercise Test and the walking distance of 6-Minute Walking Test in the exercise group were significantly better than those in the control group [(13.09±1.46) mL/kg/min *vs* (11.89±1.38) mL/kg/min, *P*=0.033; (297±46) m *vs* (243±43) m, *P*=0.041]. The average number of we-chat steps in the exercise group was also significantly better than that in the control group (4, 381±397 *vs* 3, 478±342, *P*=0.035). Forced vital capacity (FVC) and forced expiratory volume in one second (FEV_1_) in the exercise group were better than those in the control group, but the difference did not reach a statistically significant level [(1.76±0.19) L *vs* (1.60±0.28) L, *P*=0.084; (1.01±0.17) L *vs* (0.96±0.21) L, *P*=0.467].

**Conclusion:**

Postoperative exercise rehabilitation training can improve exercise endurance and daily activity ability of patients with lung cancer complicated with COPD and promote postoperative rehabilitation.

慢性阻塞性肺病（chronic obstructive pulmonary disease, COPD）是肺癌的常见合并症，影响全世界范围内45%-63%的肺癌患者，且COPD也是肺癌发生的独立危险因素之一^[1]^。合并COPD的肺癌患者围术期风险增加，术后运动耐力和肺功能较常规肺癌患者下降幅度更大^[2-4]^。即使是早期COPD患者，术后肺部并发症发生率也高于肺功能正常的非小细胞肺癌（non-small cell lung cancer, NSCLC）患者^[5]^。

疲乏和呼吸困难是两种最常见的术后肺部并发症，与术后运动耐力和肺功能下降有关^[6-9]^。在两篇系统综述中，包括运动训练在内的术后康复计划被认为能够改善未经选择的肺癌患者术后相关症状，但术后运动是否有益于合并COPD的肺癌患者尚不清楚^[10, 11]^。本研究拟探究术后运动训练对肺癌合并COPD患者运动耐力、日常活动能力和肺功能的影响。

## 1 材料与方法

### 1.1 研究对象

前瞻性纳入2020年8月5日-2021年8月25日四川大学华西医院肺癌中心行手术治疗的NSCLC患者，按纳入和排除标准筛选，并对受试者执行知情同意，最终纳入研究74例肺癌患者。纳入标准：①年龄≥18周岁；②病理诊断为原发性NSCLC；③诊断包括COPD（依据《慢性阻塞性肺疾病诊治指南》，一秒用力呼气量（forced expiratory volume in one second, FEV_1_）与用力肺活量（forced vital capacity, FVC）的比值< 0.7）；④能在指导下使用微信计步功能。排除标准：①严重的心理或精神疾病；②无法参加运动训练（如下肢功能障碍或偏瘫等）。

### 1.2 随机分组

纳入受试者由一名护士使用计算机生成随机数字表随机分为运动组和对照组，受试者不知晓自身的分组，评估测试、数据收集和统计分析人员均不知晓患者分组情况。

### 1.3 研究设计

患者在肺癌中心术前住院期间告知研究相关信息，并询问患者参加意愿。符合纳入排除条件并同意参加的患者被随机分组，并进行研究相关初次评估，一般在术前3天进行。术后第1周两组患者接受相同的术后康复治疗，术后第2周患者从肺癌中心转科到康复医学中心进行后续治疗，对照组患者按需接受常规护理，而运动组患者在此基础上开始进行术后运动训练，为期2周，运动干预结束后两组患者再次进行研究相关终点评估。鼓励所有受试者进行住院病区内活动，对所有受试者进行从初次评估到终点评估每日微信计步数的统计。

### 1.4 干预方案

术后第1周两组患者接受相同的术后康复治疗，包括早期活动、辅助咳嗽、深呼吸训练、按需吸氧和雾化治疗。对照组从第2周开始接受常规护理，包括按需吸氧、雾化，鼓励患者在住院病区内活动，运动组在此基础上开始进行术后运动训练。术后运动主要为有氧训练，运动组术后患者使用四肢联动运动30 min，每日2次，每周6天。运动强度最初设定为20%心率储备（heart rate reserve, HRR），然后逐渐增加到60%-70%心率储备，运动期间可以根据需要补充氧气。每次训练之前，受试者进行5 min的热身。根据患者的需要，30 min的训练可分为两次15 min或三次10 min，休息时间间隔最长为5 min，该运动计划改编自美国运动医学院癌症幸存者运动训练指南^[12]^。

### 1.5 结局指标

主要结局指标是运动耐力和日常活动能力的变化，次要结局指标是肺功能的变化。心肺运动试验（cardiopulmonary exercise test, CPET）和6分钟步行试验（6-minute walk test, 6MWT）用于评估运动耐力，心肺运动试验的最大耗氧量越高，6分钟步行试验的步行距离越长，说明运动耐力越好^[13, 14]^。微信计步用于比较两组患者日常活动能力，微信计步功能开启后，患者随身携带手机即可记录当日行走的全天步数。肺功能测试结果中，FVC和FEV_1_用于评估整体肺功能^[15, 16]^。

### 1.6 统计分析

在G^*^power软件（版本3.1.9.2）上计算样本量，*α*=0.05，统计功效=0.80，最小样本量为58^[17]^。考虑到20%的样本脱落率，确定74为合适的样本量，以产生显著组间统计差异。使用统计软件SPSS 23.0版进行数据处理，*Kolmogorov-Smirnov*检验用于分布模式。连续数据以平均值和标准差（Mean±SD）表示，分类数据以频率（百分比）表示。组间差异采用非配对*t*检验、*Mann-Whitney U*检验进行分析。所有统计检验均为双侧检验，*P* < 0.05为差异有统计学意义。

## 2 结果

### 2.1 基本资料

本研究中共有104例诊断为肺癌合并COPD的患者通过资格筛选，其中74例患者同意参加本项研究。随机分组后，对照组36例，运动组38例。两组共6例受试者中途退出，对照组1例受试者因心理问题退出，运动组5例受试者因术后疼痛无法训练退出，运动组其余33例患者均完成了24次训练。所有74例患者都进行了基线评估，只有68例患者进行了终点评估。

### 2.2 基线评估

两组人口统计数据如表 1所示。两组在性别、年龄、体重指数、婚姻状况、受教育年限、心血管病史、COPD病史和术前抗癌治疗方面没有明显差异。两组受试者的平均年龄为（68.9±6.1）岁，年龄最大84岁，最小55岁。24例受试（32.4%）为女性，COPD病史的平均年限为（12.3±2.4）年。表 2总结了手术特点，两组间手术类型和切除面积没有显著差异，手术过程中和术后的通气参数也没有显著差异。

**表 1:** 两组患者人口学资料和临床特征 Demographic data and clinical characteristics of two groups

	Control group (*n*=36)	Exercise group (*n*=38)	*P*
Male sex	25 (69.4%)	25 (65.8%)	0.416
Age (yr)	69.4±4.9	68.5±4.23	0.445
BMI (kg/m^2^)	23.6±2.3	24.3±1.6	0.196
Married	23 (63.9%)	26 (68.4%)	0.149
Education length (yr)	3.5 (1.6%)	3.2 (1.4%)	0.585
Diabetes mellitus	6 (16.7%)	7 (18.4%)	0.857
Coronary artery disease	5 (13.9%)	6 (15.8%)	0.785
Hypertension	18 (50.0%)	20 (52.6%)	0.636
Peripheral arterial disease	6 (16.7%)	9 (23.7%)	0.215
Preoperative treatment	5 (13.9%)	8 (21.1%)	0.131
COPD history (yr)	11.3±3.9	12.8±3.5	0.249
COPD severity			0.337
Level Ⅰ	7 (19.4%)	9 (23.7%)	
Level Ⅱ	24 (66.7%)	25 (65.8%)	
Level Ⅲ	5 (13.9%)	4 (10.5%)	
Level Ⅳ	0 (0.0%)	0 (0.0%)	
BMI: body mass index; COPD: chronic obstructive pulmonary disease; FVC: forced vital capacity; FEV_1_: forced expiration volume in one second. COPD severity: level Ⅰ refers to patients whose FVC is greater than 80% of its predicted value; level Ⅱ refers to patients whose FVC is between 50% and 80% of predicted value; level Ⅲ refers to patients whose FVC is between 30% and 50% of predicted value; level Ⅳ refers to patients whose FVC is less than 30% of predicted value.			

**表 2:** 两组手术特征和机械通气数据 Surgical characteristics and mechanical ventilation data of two groups

	Control group (*n*=36)	Exercise group (*n*=38)	*P*
Resection sites			0.371
Segmentectomy	9 (25.0%)	13 (34.2%)	
Lobectomy	26 (72.2%)	24 (63.2%)	
Pneumonectomy	1 (2.8%)	1 (2.6%)	
Type of surgery			0.529
VATS	24 (66.7%)	28 (73.7%)	
Thoracotomy	12 (33.3%)	10 (26.3%)	
Duration of surgery (min)	187±51	195±47	0.324
Duration of anesthesia (min)	273±72	285±80	0.357
VT during TLV (mL/kg PBW)	7.9±1.8	8.3±1.7	0.222
PEEP during TLV (cmH_2_O)	5±1	5±2	0.851
FIO_2_ during TLV (%)	54±11	56±14	0.463
VT during OLV (mL/kg PBW)	6.0±1.5	6.3±1.8	0.357
PEEP during OLV (cmH_2_O)	6±1	6±2	0.683
FIO_2_ during OLV (%)	63±16	67±21	0.190
PaO_2_/FIO_2_ on POD1 (%)	34±15	31±17	0.236
VT: tidal volume; TLV: two-lung ventilation; PBW: predicted body weight; PEEP: positive end-expiratory pressure; FIO_2_: fraction of inspiratory oxygen; OLV: one-lung ventilation; POD1: first postoperative day; PaO_2_/FIO_2_: ratio of partial oxygen pressure to inspiratory fraction of oxygen. TLV data obtained in 10 min after beginning of TLV mode, and OLV data obtained in 10 min after beginning of OLV mode.			

### 2.3 主要结局指标

从基线到终点的功能状态见表 3，微信计步数据见表 4，肺功能测试结果见表 5。与基线评估相比，两组的运动耐力参数均有所下降。但在手术和干预方案后，运动组的心肺运动试验中的最大耗氧量和6分钟步行试验中的步行距离均显著优于对照组[(13.09±1.46) mL/kg/min *vs* (11.89±1.38) mL/kg/min, *P*=0.033; (297±46) m *vs* (243±43) m, *P*=0.041]。两组患者日常活动能力在监测周期的多数天数内无明显差异，但在术后第17、20和21天，运动组微信计步数显著高于对照组。

**表 3:** 两组基线运动耐力和肺功能 Functional status and pulmonary function test of baseline assessment of two groups

	Control group (*n*=36)	Exercise group (*n*=38)	*P*
CPET (mL/kg/min)	16.13±1.69	16.72±1.86	0.370
6MWT (m)	351±52	366±57	0.348
FVC (L)	2.16±0.49	2.02±0.35	0.160
FEV_1_ (L)	1.32±0.27	1.24±0.21	0.761
CPET: cardiopulmonary exercise test. 6MWT: 6-minute walk test.			

**表 4:** 两组患者微信计步数 Step-counting by We-chat Sport App between groups

Date	3 pre^*^	2 pre	1 pre	Due surgery	1 post^*^	2 post	3 post	4 post	5 post
Control group (*n*=36)	6, 249±549	5, 141±492	5, 980±533	33±4	6±1	14±2	43±4	138±11	292±19
Exercise group (*n*=38)	6, 322±592	5, 738±518	5, 716±527	38±4	9±1	17±2	57±5	152±13	275±20
*P*	0.762	0.892	0.620	0.829	0.591	0.835	0.709	0.650	0.903
Date	6 post	7 post	8 post	9 post		10 post	11 post	12 post	13 post
Control group (*n*=36)	554±49	738±77	942±93	1, 189±109		1, 431±137	1, 705±172	1, 999±192	2, 298±217
Exercise group (*n*=38)	594±52	668±70	998±89	1, 293±121		1, 532±149	1, 621±168	1, 857±187	2, 201±224
*P*	0.761	0.542	0.670	0.519		0.731	0.201	0.379	0.752
Date	14 post	15 post	16 post	17 post		18 post	19 post	20 post	21 post
Control group (*n*=36)	2, 505±248	2, 797±270	2, 990±287	3, 184±321		3, 393±324	3, 495±338	3, 590±341	3, 478±342
Exercise group (*n*=38)	2, 636±256	3, 059±289	3, 308±300	3, 779±349		3, 649±349	3, 703±369	3, 947±378	4, 381±397
*P*	0.184	0.107	0.222	0.046		0.119	0.093	0.021	0.035
3 pre^*^: 3^rd^ day pre-surgery; 1 post^*^: 1^st^ day post-surgery.									

**表 5:** 两组终点运动耐力和肺功能 Functional status and result of pulmonary function test of endpoint assessment of two groups

Index	Control group (*n*=35)	Exercise group (*n*=33)	*P*
CPET (mL/kg/min)	11.89±1.38	13.09±1.46	0.033
6MWT (m)	243±43	297±46	0.041
FVC (L)	1.60±0.28	1.76±0.19	0.084
FEV_1_ (L)	0.96±0.21	1.01±0.17	0.467
one case in the control group withdrew due to psychological problems; five cases in the exercise group withdrew due to postoperative pain.			

### 2.4 次要结局指标

两组患者术后肺功能测试也出现下降。运动组的FVC和FEV_1_仍优于对照组，但差异未达到统计学显著水平[(1.76±0.19) L *vs* (1.60±0.28) L, *P*=0.084; (1.01±0.17) L *vs* (0.96±0.21) L, *P*=0.467]。

## 3 讨论

全世界范围内45%-63%的肺癌患者受到COPD的影响^[18]^，经手术切除治疗后患者最大摄氧量平均降低15%-30%，而合并COPD的患者较常规患者运动耐力降低幅度更大^[19, 20]^。本研究中两组患者的心肺运动试验和6分钟步行试验结果术后较术前均有所下降，但经过术后运动训练干预，运动组患者两项运动耐力测试结果的下降幅度显著低于对照组。在我们的研究之前，Edvardsen等^[21]^报道了高强度耐力和力量训练可以在临床上显著改善峰值摄氧量和功能适应性。Salhi等^[22]^的研究证明了肺癌患者的康复计划能够显著提高运动能力和肌肉强度。本研究扩展了此前研究的患者群体，肺癌合并COPD患者也可以从术后运动训练中获益，提高运动耐力。

肺癌手术对上肢活动的影响高于下肢活动，多数术后患者早期以慢走、快走和慢跑作为主要术后运动方式，本研究创新的采用微信计步数量对患者日常活动能力进行测量对比。两组患者在监测周期内多数天数无明显差异，在术后第17、20和21天，运动组步数显著高于对照组，表明术后运动训练对日常活动能力的恢复有促进作用。

运动组肺功能测试的结果也优于对照组，但未达到统计学显著水平，这可能是因为本研究样本容量小，不足以产生显著的组间差距。研究结果与另一项研究^[23]^肺癌患者术后运动加吸气肌训练效果的临床试验一致，该研究中干预后呼吸肌强度没有明显改善，但也呈现有利于运动组的趋势。

本研究也存在一些局限性。首先，由于研究设计时考虑到不能将所有同组患者置于同一住院病区（否则研究者便可知晓受试者分组情况），不同组受试者可能住在同一病区，患者之间的交流模仿可能导致组间差异减小；第二，微信步数计量只能大致反应患者日常活动量，在准确度方面也存在一定缺陷，如果患者更多通过太极或深蹲这样的方式训练，步数记录就不会有改变；此外患者步幅差异也是造成步数差异的因素^[24]^。

肺癌合并COPD患者术后恢复周期较单纯肺癌患者更长，术后最常见的躯体症状是运动耐力下降和运动后呼吸困难^[25]^。本研究表明监督下进行有计划的术后运动训练可以加快肺癌合并COPD患者术后运动耐力和日常活动能力的恢复，为此类患者的多学科综合管理提供新的选择。
